# Immature B cell homing shapes human lymphoid tissue structure and function

**DOI:** 10.1084/jem.20240085

**Published:** 2024-08-02

**Authors:** Jo Spencer, Chiara Dionisi

**Affiliations:** 1 Peter Gorer Department of Immunobiology, School of Immunology and Microbial Sciences, King’s College London, London, UK

## Abstract

Shortly after the emergence of newly formed human B cells from bone marrow as transitional cells, they diverge along two developmental pathways that can be distinguished by the level of IgM they express and migratory biases. Here, we propose that differential tissue homing of immature B cell subsets contributes to human lymphoid tissue structure and function.

## Teaser

After leaving the bone marrow, transitional B cells split into subsets with a biased expression of homing receptors accompanied by different levels of surface IgM. Here, Spencer and Dionisi discuss how this could contribute to human lymphoid tissue content and function.

## Bifurcation in systemic human B cell maturation

Human B cells mature systemically following bone marrow egress. This progress cannot be mapped onto the biology of mouse B cells that differ from human B cells in many lineage features and maturation dynamics and thus must be considered directly by taking information from the multiple sample sites and data types available (Gibbons and Spencer, 2011; Steiniger, 2015; Weisel et al., 2022).

Human bone marrow emigrants progress from early transitional (T) 1 B cells to T2 B cells. Maturation is gradual and visualized by loss of CD10, CD24, and CD38, and gain of CD21 along a differentiation continuum (Palanichamy et al., 2009). The presence of stable percentages of T1 and T2 cells in human blood and the sequential repopulation of these compartments following B cell depletion therapies or ablation prior to hematopoietic stem cell transplantations are consistent with the idea that bone marrow egress and maturation are continuous and that observed percentages of cells in blood reflect a dynamic steady state (Bemark, 2015; Bemark et al., 2012; Palanichamy et al., 2009).

By the T2 stage of B cell development, two parallel populations emerge that resemble each other in terms of the markers of maturation described above that define T2 cells, but which can be distinguished from each other by the amount of surface IgM they express measured by either flow cytometry or cell surface staining with oligonucleotide-labeled antibodies in single-cell RNA-sequencing analysis (Tull et al., 2021). They cannot be distinguished by the amount of transcript encoding the IGHμ chain that appears independent of protein expression density (Tull et al., 2021).

**Figure 1.**
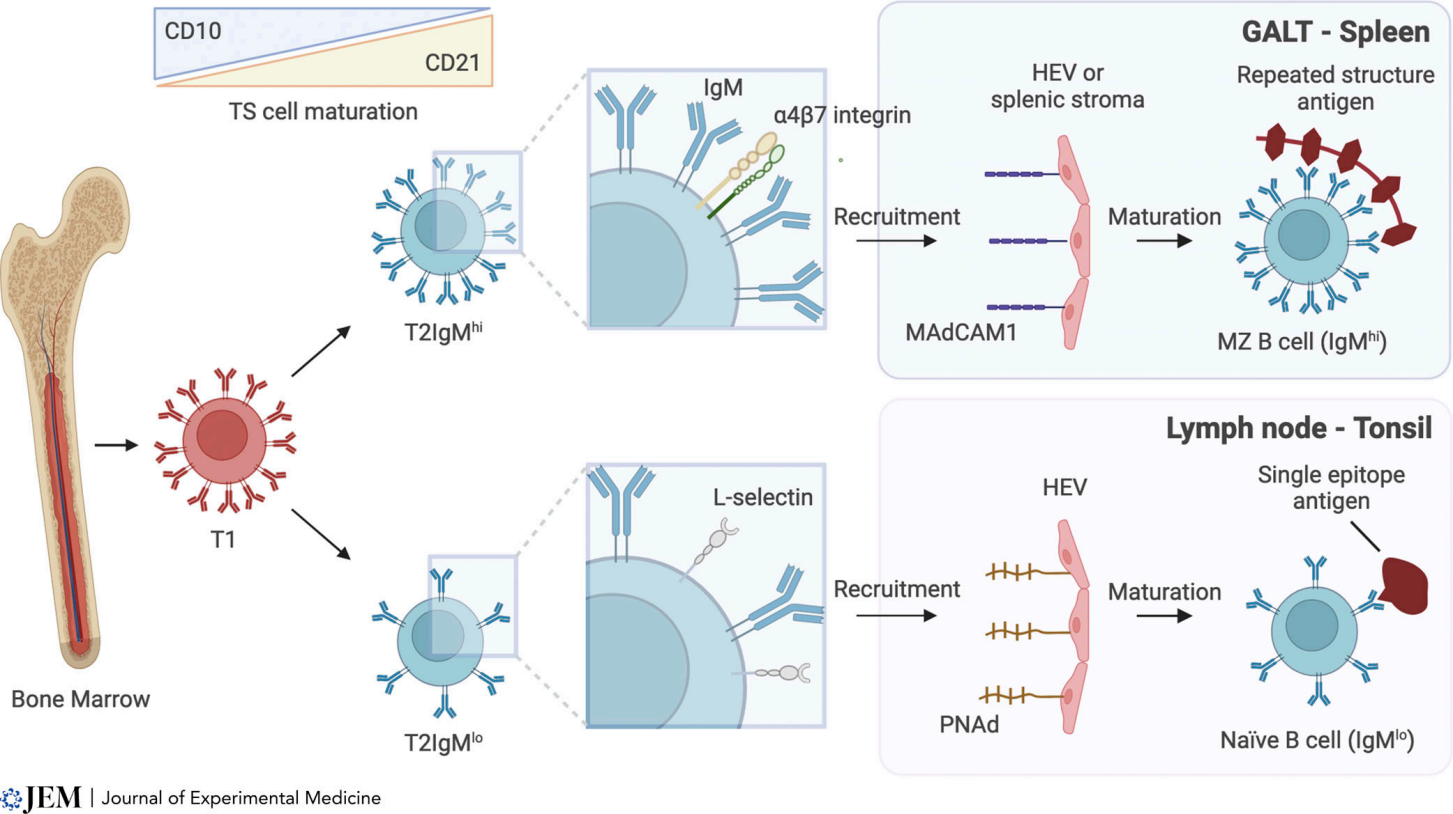
**Recruitment of immature B cells to lymphoid tissues.** B cells exit the bone marrow as T1 B cells, expressing high levels of IgM and CD10. Their transition to T2 cells is characterized by a reduction in CD10 expression and gain of CD21, with two distinct developmental pathways becoming apparent. T2 IgM^hi^ cells selectively express α4β7 integrin, which mediates homing to GALT and spleen through binding to its cognate receptor MAdCAM-1. T2 IgM^lo^ cells express relatively more L-selectin and are thus enriched in their potential to enter lymphoid tissues expressing its ligand PNAd, including lymph nodes and tonsils. We propose that T2 IgM^hi^ and T2 IgM^lo^ cells mature into MZB and naïve B cells, respectively. Higher receptor surface expression may enable IgM^hi^ cells to respond to multivalent repeated subunit antigens that drive TI responses. IgM^lo^ cells are instead adapted to T-dependent responses to single epitope antigens.

Features that discriminate between IgM^hi^ and IgM^lo^ T2 B cells reliably across datasets and methodologies include differential expression of receptors associated with tissue site–specific homing (Tull et al., 2021) (Fig. 1). IgM^hi^ T2 cells express relatively higher expression of integrin α4β7 that mediates homing into organized gut-associated lymphoid tissue (GALT) and the diffuse immune infiltrate of the intestinal lamina propria through binding to mucosal vascular addressin cell adhesion molecule 1 (MAdCAM1) expressed by intestinal endothelium (Berlin et al., 1993; Briskin et al., 1997; Streeter et al., 1988a). Expressed α4β7 also mediates retention in the splenic marginal zone mediated by MAdCAM1 expressed by marginal reticular cells in adults (Magri et al., 2014; Steiniger et al., 2022). In contrast, the IgM^lo^ T2 cells express relatively higher expression of L-selectin (CD62L) that binds to the peripheral node addressin (PNAd), mediating recruitment into peripheral lymph node or tonsil (Michie et al., 1993; Streeter et al., 1988b). Intriguingly, the expression of PNAd and MAdCAM1 by high endothelial cells throughout the body in adults is inversely related, except for the mesenteric nodes that express both (Streeter et al., 1988b). This suggests that maturing T2 cells also migrate in a biased way into either the GALT and spleen axis, or the peripheral node and tonsil in adults. In addition to CD62L, IgM^lo^ cells also express relatively high CCR7, which binds CCL19, and CCL21, which guides extravasated cells to the T cell zones of lymphoid tissues (Sallusto et al., 1999). Both CD62L and CCR7 are associated with lymphocyte recirculation (Baekkevold et al., 2001; Watson and Bradley, 1998).

## Can we observe transitional cell subsets that have extravasated in different tissue locations?

The opposing dominant expression of either α4β7 integrin by T2IgM^hi^ or CD62L with CCR7 by T2IgM^lo^ cells respectively, and the relative lack of expression of either receptor by T1 cells suggest that they tend to migrate along different paths. The T1 subset has been observed in spleen in humans as in mice (Palanichamy et al., 2009; Pillai and Cariappa, 2009). In contrast, T1 cells are excluded from GALT and remain present in blood in the liver that arrives via the portal vein, whilst the T2IgM^hi^ cells are depleted from these isolates by prior selective extravasation into the GALT (Tull et al., 2021; Vossenkämper et al., 2013). Therefore, although GALT and spleen share features related to α4β7 expression and potential T2IgM^hi^ recruitment (Kraal et al., 1995), only the spleen recruits T1 cells. The mechanism of T1 extravasation into human spleen and its consequences are not known, though the observations are consistent with a role for spleen in early B cell fate decisions as in mice, potentially involving AKT activity in both species (Cerutti et al., 2013; Cox et al., 2023; Pillai and Cariappa, 2009).

Selective presence of T2IgM^hi^ but not T1 or T2IgM^lo^ cells in GALT has been directly observed by flow cytometry and mass cytometry, supporting the notion of divergent tissue migratory routes of T2 cells (Tull et al., 2021). Supporting this, CD1c-expressing B cells, a feature of T2IgM^hi^ cells, have also been observed in fetal intestine (Guo et al., 2023).

Once in GALT in adults, T2 cells become activated as evidenced by their expression of CD69 and CD80, and phosphorylation of BTK, Syk, and ERK, which are not features of T2 cells in paired blood (Tull et al., 2021; Vossenkämper et al., 2013). The activation of T2 cells in GALT has been proposed to be an early step toward their development into marginal zone B (MZB) cells via an intermediate MZB precursor stage that is likely to be driven by the GALT microenvironment that is enriched in bacterial antigens and involves the acquisition of mutations in GALT germinal centers (GC). Consistent with this, T2IgM^hi^ but not T2IgM^lo^ cells can be stimulated to differentiate toward MZB precursor development via engagement of TLR9 (Tull et al., 2021), though other signals are likely to be involved that could include the Notch2 ligand Delta-like 1, other stromal signals, and interactions with innate lymphoid cells (ILC) (Descatoire et al., 2014; Magri et al., 2014). The hypothesis that MZB mature and diversify their IGHV genes in GALT GC is supported by analysis of the human B cells’ response to the Pneumovax vaccine, which is composed of capsular polysaccharides and is considered a model T cell–independent (TI) type 2 (TI2) antigen. The responding B cells include MZB cells that show pre-existing antibody specificity for gut bacteria, encoded by a receptor prediversified by somatic hypermutations consistent with a role for GALT GC (Weller et al., 2023).

## Functional features of T2 cell subsets mirror properties of tissues that their homing receptors guide them to

The transcriptomic features of T2IgM^lo^ cells are consistent with the potential to undergo T-dependent B cell responses. Their relatively high expression of IL4R is consistent with the ability to interact with T cells to support the GC response (Chakma and Good-Jacobson, 2023; Chevrier et al., 2017; Possamaï et al., 2021; Robinson et al., 2019). T2IgM^lo^ cells are also characterized by enriched expression of KLF2 that is associated with follicular B cell survival, migration, and BCR-mediated signaling and response to specific antigens, all of which are compromised when KLF2 is knocked out in mice (Hart et al., 2011; Hoek et al., 2010; Tull et al., 2021; Winkelmann et al., 2011). The low level of BCR expressed by T2IgM^lo^ cells could also bias against recognition of TI2 antigens but favor higher affinity recognition of single epitopes antigens. It is possible that T2IgM^lo^ cells preferentially develop into canonical naïve B cells; indeed, this has been proposed (Morgan and Tergaonkar, 2022).

Features associated with the functional capacity of T2IgM^hi^ cells include the expression of CD1c that mediates lipid presentation (Roy et al., 2014; Tull et al., 2021). Expression of CD1c is a feature of MZB cells, alongside expression of CD27 and IgD (Weill et al., 2009). T2IgM^hi^ also acquire the expression of TNF receptor superfamily member 13B (TNFRSF13B, also known as transmembrane activator and calcium modulator and cyclophilin ligand interactor [TACI]) along their described maturation trajectory towards MZB cells (Tull et al., 2021). TACI is known to support TI B cell responses and is important for efficient TI IgA responses in human GALT (Grasset et al., 2020; Mantchev et al., 2007; von Bülow et al., 2001). A high density of IgM expression could favor activation in response to the multivalent repeated subunit antigens that drive innate-like and TI B cell responses (Fig. 1).

GALT and spleen that recruit or retain cells by mechanisms that involve MAdCAM-1 and that are both associated with TI B cell responses in humans have microanatomical marginal zones that contain MZB and memory B cells (Siu et al., 2022; Spencer et al., 1985; Weill et al., 2009; Zhao et al., 2018). In contrast, other lymphoid tissues such as tonsils and lymph nodes can contain MZB and memory cells, but the dominant B cell profile is of naïve B cells that surround the GC, if a GC is present (Zhao et al., 2018). MZB and memory cells in lymph node and tonsil tend to localize in the subcapsular sinus or the pseudostratified epithelium, respectively, if present (Lazzi et al., 2006; Morente et al., 1992).

## Do immature B cell subsets contribute to the functional potential of the lymphoid tissue they migrate into?

**Figure 2.**
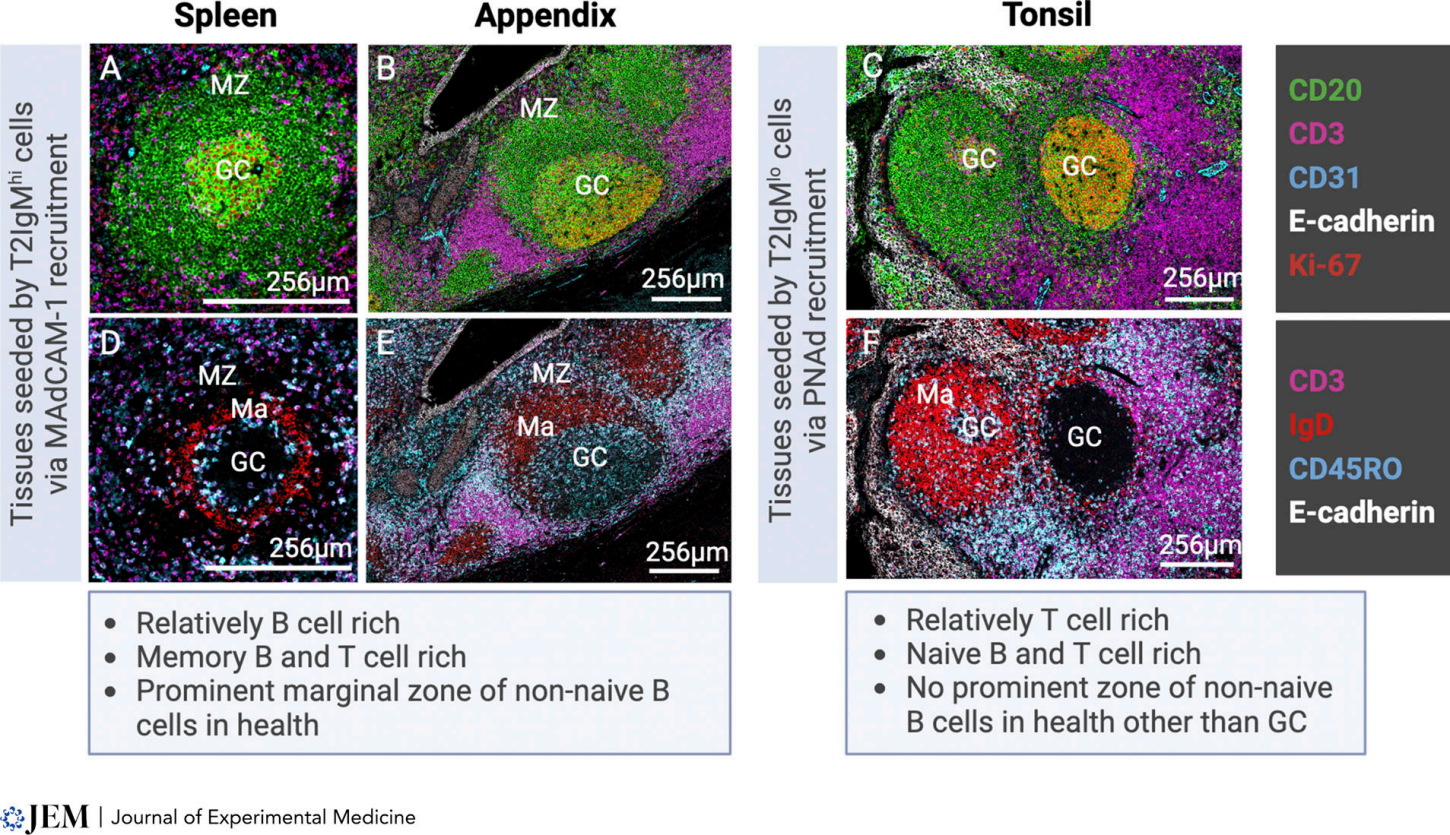
**Features of lymphoid tissues with contrasting recruitment biases. (A–F)** Images in A–F were acquired by imaging mass cytometry analysis of normal human spleen (A and D), appendix (B and E), and tonsils (C and F) to illustrate the previously described features of tissues seeded by T2 IgM^hi^ (A, B, D, and E) or T2 IgM^lo^ cells (C and F). Images in A, B, and C illustrate B cells (CD20^+^, green), T cells (CD3^+^, magenta), epithelium (E-cadherin^+^, white), and endothelium (CD31^+^, cyan). GALT (A) and spleen (B) are relatively rich in B cells in comparison with tonsils (C) and have a well-defined microanatomical marginal zone on the periphery of the B cell follicle. In each case, the GC contains proliferating cells (Ki-67^+^, red). Tonsils have a relatively greater abundance of T cells compared with GALT or splenic white pulp (C compared to A and B). Lymphocyte subtypes in tonsils are biased toward naïve cells, as indicated by fewer T cells expressing CD45RO (cyan) and relatively more naïve B cells expressing IgD (red) compared with GALT and spleen (F compared to D and E). MZ = marginal zone; Ma = mantle zone of naïve cells.

The observed steady state of early systemic human B cell maturation reflects a constant stream of B cells from bone marrow to blood, to spleen (T1) and subsequently biased migration of subsets of T2 cells toward different tissue locations. Here, we suggest that the influx of T2 cells with varying densities of BCR into tissues can be followed by maturation from T2IgM^hi^ to MZB and T2IgM^lo^ to naïve B cells, and that this contributes to the functional potential of recipient tissues and the associated recirculating pool (Fig. 2).

We propose that it is not a coincidence that T2IgM^lo^ cells that have higher expression of IL4R and KLF2 and low B cell receptor density (Tull et al., 2021) and that have been proposed to be precursors of naïve cells (Morgan and Tergaonkar, 2022) preferentially migrate into lymph nodes and tonsils that are enriched in naïve cells compared with GALT and spleen (Vidal-Rubio et al., 2001; Zhao et al., 2018) (Fig. 2). An abundance of naïve cells would increase repertoire diversity and enhance the probability of an epitope encountering a complementary receptor. Lymph nodes are also relatively T cell enriched, enhancing the chances of cognate B cell interaction and the potential for generation of high-affinity B cell responses (Langeveld et al., 2006; Scott et al., 2018; Tedla et al., 1999) (Fig. 2). Lymph nodes classically drain sites commonly selected for vaccination due to their relatively high effectiveness in response (Miyasaka, 2022).

T2IgM^hi^ cells which have higher expressions of CD1c, TACI, and higher receptor density that can favor immune responses to TI2 antigens with repeating subunit structures preferentially enter GALT and spleen, which are both relatively enriched in B cells and are each associated with TI B cell responses (Amlot et al., 1985; Grasset et al., 2020; Tull et al., 2021; Weller et al., 2023). Naïve cells are relatively sparse in GALT and spleen (Fig. 2) that are each biased toward more innate-like B cell responses. Although GALT contains chronically active GC, B cell entry into such GC is not necessarily thought to be dependent on cognate interactions with T cells. Immune responses to carbohydrate epitopes, for example, may involve conjugates, but could also involve co-processed epitopes, T cell contact, T cell–derived cytokines, or be completely T cell independent, as reviewed elsewhere (Bemark et al., 2024; Kabbert et al., 2020). Thus, GALT GC are not solely involved in the generation of high-affinity responses to protein antigens. GALT is notoriously difficult to vaccinate to generate protective intestinal responses. Though there are some notable successes (Blume and Geesink, 2000; Holmgren, 2021), the wide range of vaccines for intramuscular injection that drive high-affinity protective responses are not matched quantitatively by the available licensed mucosal vaccines (Baker et al., 2022; Lavelle and Ward, 2022). This is despite considerable effort and excellent progress in the development of antigen delivery systems and conjugates (Kwong et al., 2023) and the known ability of intestinal plasma cells to be long-lived (Landsverk et al., 2017). We propose that the structures and cells in GALT are biased toward innate-like or unconventional responses that are highly effective in controlling pathogens but are not necessarily geared toward the generation of high-affinity responses in adulthood and that the subtype of immature B cell recruited has features that contribute to this on maturation.

## Conclusion

Lymphoid tissues vary in terms of routes of antigen entry and the type and quantity of the antigen experienced. While acknowledging that the factors that determine the features of the immune response are complex, we propose that the recruitment of precursors of B cells with contrasting functional biases makes an unanticipated contribution to this.
